# Ragomics‐Meta: An open‐source, scalable, reproducible, and extensible multi‐omics analysis workflow

**DOI:** 10.1002/imo2.70121

**Published:** 2026-07-17

**Authors:** Zeping Gui, Huidong Su, Yuhao Chen, Jixing Yuan, Yexiu Gao, Ze Yu, Jing Zhang, Qingyi Zhu, Min Gu, Mulong Du, Zijie Wang, Ming Chen

**Affiliations:** ^1^ Department of Urology, Jiangsu Key Laboratory of Urological Disease Prevention and Treatment The Second Affiliated Hospital of Nanjing Medical University, Nanjing Medical University Nanjing China; ^2^ Research and Development Department RageneBio Inc. Verona New Jersey USA; ^3^ Department of Paediatrics and Adolescent Medicine, LKS Faculty of Medicine The University of Hong Kong Hong Kong SAR China; ^4^ Department of Biostatistics, Center for Global Health, School of Public Health Nanjing Medical University Nanjing China

## Abstract

Ragomics‐Meta is an open‐source, scalable, and reproducible multi‐omics workflow for metabolism‐centered analysis (https://www.raganetwork.com/). It integrates scRNA/snRNA‐seq, spatial transcriptomics, scATAC‐seq, and metabolomics modules to support quality control, dimensionality reduction, clustering, differential expression and accessibility analyses, pathway activity scoring, cell−cell communication inference, transcription‐factor network analysis, and spatial metabolism exploration. By incorporating established tools such as Scanpy, Seurat, Squidpy, COMMOT, SnapATAC2, MEBOCOST, scMetabolism, AUCell, and a curated gutMGene‐based resource, Ragomics‐Meta enables systematic characterization of tumor metabolic programs and host−microbiome interactions. AI‐assisted interpretation further facilitates biological annotation and hypothesis generation, providing a user‐friendly framework for integrative single‐cell and spatial multi‐omics analysis.

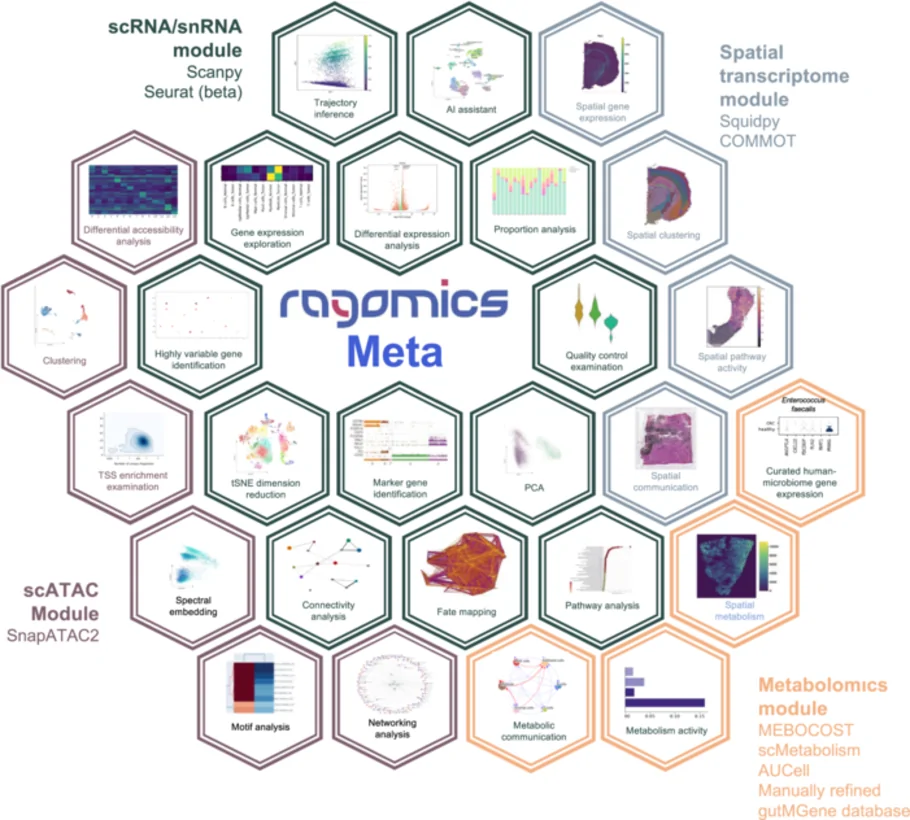

To the editor,

Tumor metabolism is increasingly recognized as a central determinant of cancer progression, therapeutic response, and microenvironmental adaptation, yet its spatial and cellular heterogeneity remains difficult to resolve across modalities [1]. Recent advances in single‐cell transcriptomics, chromatin accessibility profiling, spatial transcriptomics, and metabolomics have greatly expanded our ability to interrogate tumor ecosystems. However, these data are often analyzed in isolation, limiting the reconstruction of coordinated metabolic programs and microbe‐host interactions within tumors [2, 3]. Although existing tools are powerful for individual modalities, they rarely provide an integrated, reproducible, and user‐friendly framework for metabolism‐centered multi‐omics analysis. This fragmentation is particularly problematic in cancer metabolism, where biologically meaningful signals often emerge only through the joint interpretation of transcriptional, epigenetic, spatial, and metabolite‐mediated communication layers. In addition, complex analytical pipelines, inconsistent parameter settings, and limited interpretability continue to hinder robust cross‐study comparison and broader practical adoption.

To address these challenges, we developed Ragomics‐Meta, an open‐source, scalable, and extensible workflow for the integrative analysis of single‐cell RNA sequencing (scRNA‐seq), single‐cell assay for transposase‐accessible chromatin using sequencing (scATAC‐seq), spatial transcriptomics, and single‐cell metabolomics data. Rather than functioning as a simple collection of existing tools, Ragomics‐Meta provides a unified and reproducible analytical framework that supports end‐to‐end analysis, cross‐omics integration, and metabolism‐centered biological interpretation. Its modular architecture further incorporates AI‐assisted components to improve reproducibility, facilitate cell annotation, accelerate biological interpretation, and enable systematic dissection of tumor metabolic programs and tumor–microbiome interactions in clinically relevant settings. We evaluated Ragomics‐Meta in acute lymphoblastic leukemia and applied it to colorectal cancer (CRC) to demonstrate its utility for multi‐omics integration and metabolism‐focused discovery [4, 5, 6, 7, 8].

## 1 INTEGRATED WORKFLOW ARCHITECTURE AND FUNCTIONAL CAPABILITIES OF RAGOMICS‐META

Ragomics‐Meta is a comprehensive workflow for integrating spatial multi‐omics with single‐cell multi‐omics to enable deeper biological insight. Each uploaded dataset is handled as an independent analysis task, allowing users to keep data private or share it publicly (Figure 1A). This task‐based design simplifies data organization, especially when managing many projects, and improves overall processing efficiency. For every task, Ragomics‐Meta provides detailed guidance, supporting smooth navigation of the workflow and transparent progress monitoring (Figure 1B).

**Figure 1:**
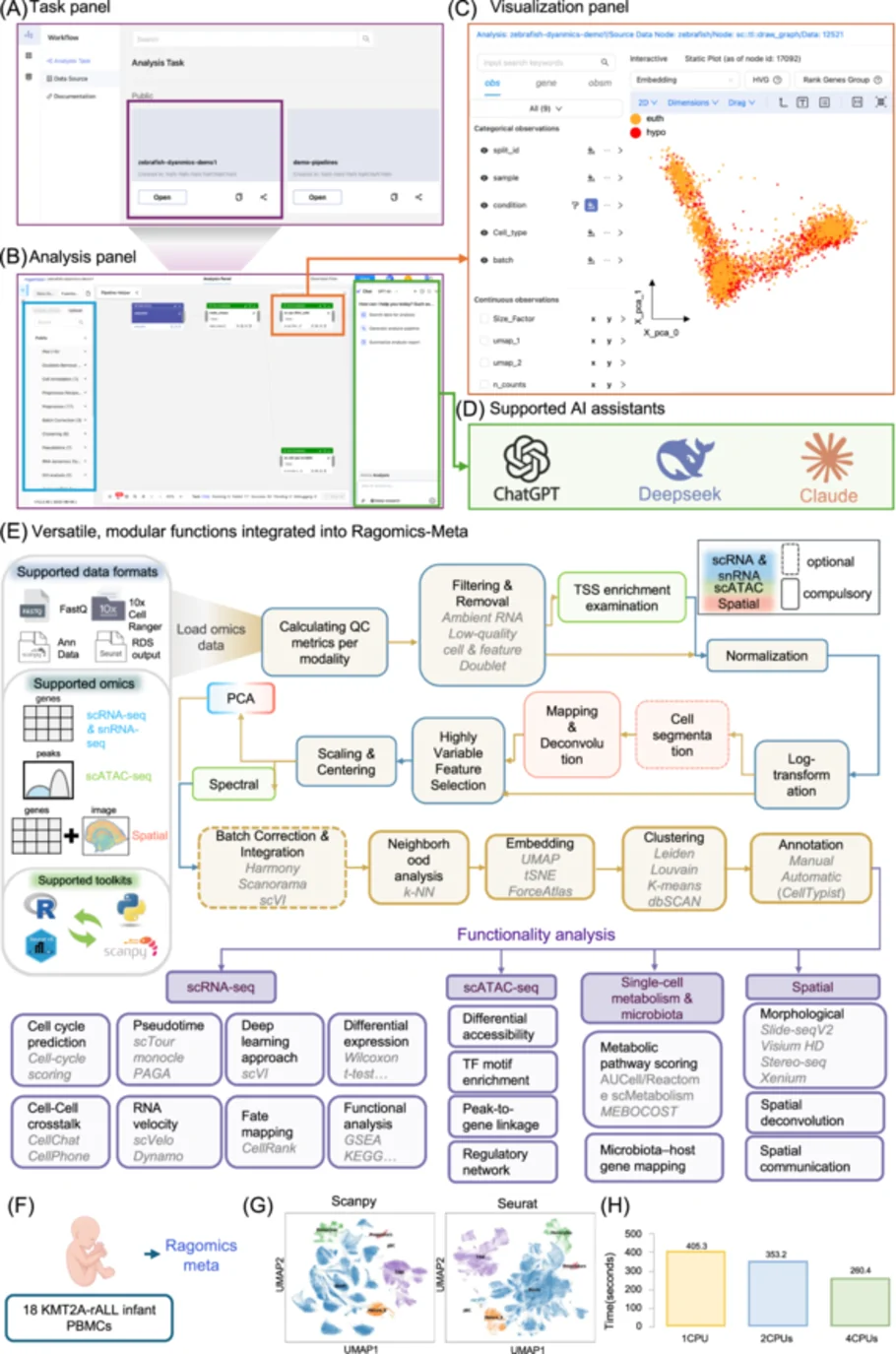
Ragomics‐Meta: A Modular Workflow for Integrative Single‐Cell Multi‐Omics Analysis. (A) The homepage of the Ragomics‐Meta. (B) Overview of the Ragomics‐Meta analysis panel, including functional modalities, LLM‐based AI chatbot, and interactive visualization panel for each task. (C) An interactive visualization panel enabling dynamic exploration by cell metadata or user‐defined genes of interest. (D) Support for multiple AI chatbots to enhance data interpretation and downstream analyses. (E) Versatile, modular functions designed to accelerate standard single‐cell analysis tasks. (F) Validation of Ragomics‐Meta's scalability and reproducibility using scRNA‐seq data from ALL. (G) Comparative UMAP embeddings generated via the Scanpy‐Zheng recipe (left) versus the Seurat recipe (right). (H) Benchmark of preprocessing runtimes under diverse computational resource allocations (asterisk denotes a configuration comparable to a typical local server).

Ragomics‐Meta also includes an interactive visualization module for intuitive exploration of data structure (Figure 1C). The module supports diverse analytical needs by enabling users to switch between dimensionality‐reduction methods (e.g., UMAP, t‐SNE, diffusion maps), toggle between 2D and 3D views, customize plot elements (e.g., legend position, axis scaling), and interactively select cell populations for targeted downstream analyses. Together, these features improve interpretability, flexibility, and analytical efficiency.

To further streamline interpretation and downstream exploration, Ragomics‐Meta supports multiple LLM‐based AI assistants, including ChatGPT, DeepSeek, and Claude (Figure 1D). In addition, it integrates 100+ functions from widely used, state‐of‐the‐art tools, such as Scanpy, Seurat, SnapATAC2, Squidpy, COMMOT, MEBOCOST, and scMetabolism, covering nearly all core tasks in spatial and single‐cell omics (Figure 1E). Each function is accompanied by clear documentation and organized into modular components, including preprocessing, spatial transcriptomics, spatial metabolism, scRNA/snRNA‐seq, scATAC‐seq, and static visualization. The curated reference resources supporting these modules, including human−microbiome interaction genes, KEGG metabolism‐associated gene sets, and Reactome metabolism‐associated gene sets, are provided in Supporting Information S1: Tables S1–S3. This modular design makes Ragomics‐Meta both robust for routine analyses and readily extensible for new omics workflows.

## 2 VALIDATION OF SCALABILITY AND REPRODUCIBILITY OF RAGOMICS‐META USING scRNA‐SEQ DATA FROM LYMPHOBLASTIC LEUKEMIA (ALL)

We first evaluated Ragomics‐Meta's robustness using a large‐scale scRNA‐seq dataset from Chen et al. profiling 18 infants with KMT2A‐rearranged acute ALL (128,588 cells; 20,043 genes). Ragomics‐Meta faithfully recapitulated the published data (Figure 1F), demonstrating reliable handling and visualization of high‐dimensional data at scale.

We next highlighted the toolkit's modular preprocessing design and computational scalability by running two Ragomics function blocks in parallel: a Python‐based Scanpy‐Zheng workflow and an R‐based Seurat pipeline. Despite differences in implementation, both workflows preserved the global data geometry in UMAP space (Figure 1G) and completed end‐to‐end preprocessing in under 5 min when multi‐central processing unit (CPU) resources were enabled. Performance benchmarking across CPU allocations further showed that using four CPUs reduced preprocessing time by >35% relative to a single‐CPU local server configuration (Figure 1H), substantially accelerating large‐scale single‐cell analyses.

## 3 INTEGRATED SINGLE‐CELL AND SPATIAL MULTI‐OMICS REVEAL IRON‐CENTERED EPIGENETIC AND METABOLIC CROSSTALK IN CRC BY RAGOMICS‐META

To demonstrate Ragomics‐Meta in a real‐world scenario, we applied it to the scRNA‐seq dataset from Lee et al. (10 samples from two patients; CRC and matched normal; Figure 2A) to characterize host gut metabolic programs in CRC [9]. Following quality control (Supporting Information S1: Figure S1A,B) and Harmony‐based batch correction (Figure 2B), Leiden clustering (resolution = 0.5) identified 15 clusters. Cell types were assigned using canonical markers and supported by PAGA connectivity analysis (Supporting Information S1: Figure S1C,D). We further benchmarked three annotation strategies, manual curation, large language model (LLM)‐assisted labeling, and CellTypist automated annotation (Figure 2C), and incorporated CellTypist to generate automated annotations.

**Figure 2:**
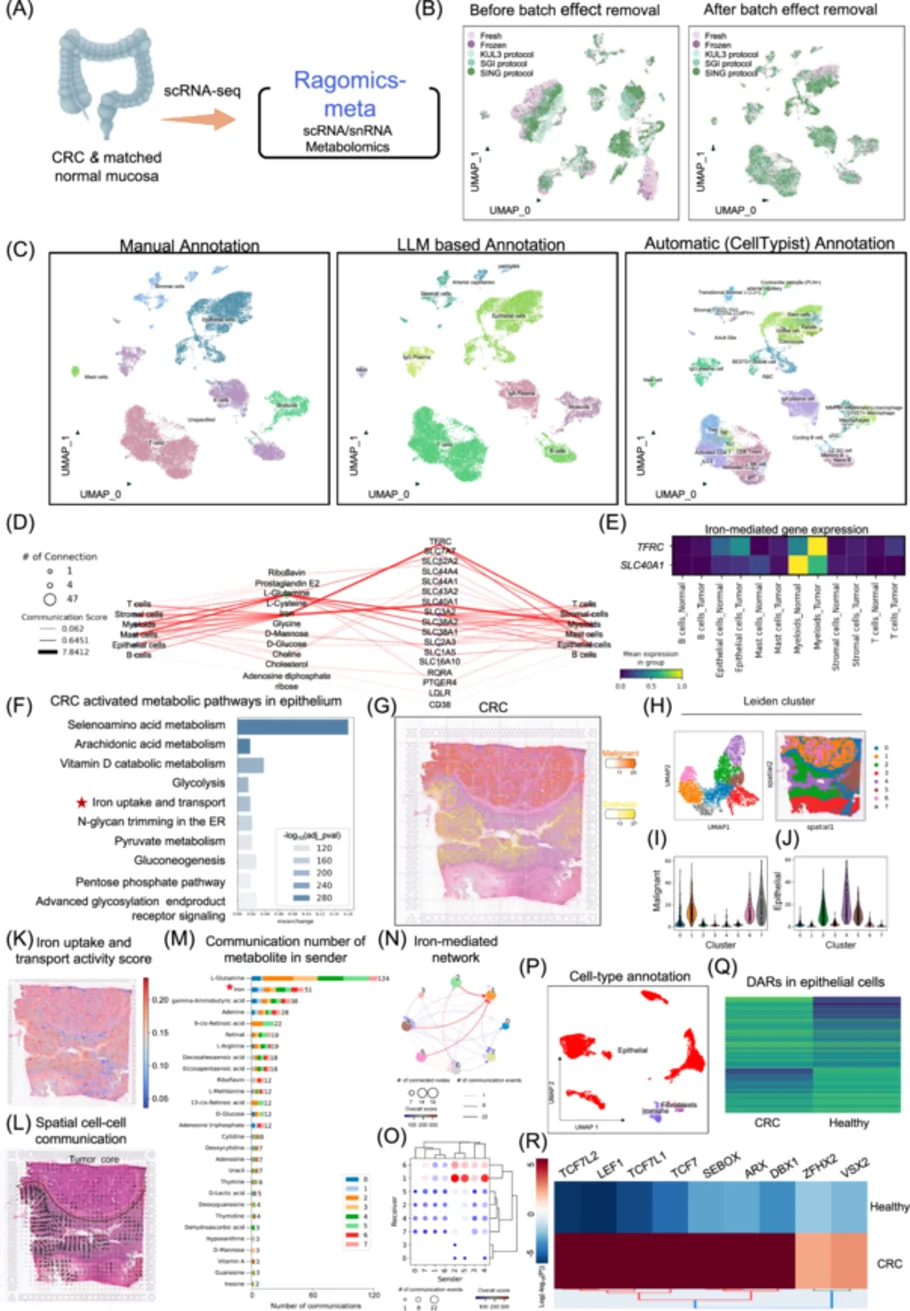
Comprehensive single‐cell and spatial multi‐omics profiling of colorectal cancer. (A) Schematic of CRC scRNA‐seq data collection and processing. (B) UMAP embeddings before and after batch correction. (C) UMAP comparison of cell‐type annotations by manual curation (left), LLM‐assistant (middle) and CellTypist auto‐annotation (right). (D) River plot of metabolite‐mediated signaling pathways from sender to receiver cells. (E) Matrix plot of *TFRC* and *SLC40A1* expression in CRC versus healthy samples across all cell types. (F) Bar plot showing the top 10 CRC‐associated differential metabolic pathways in epithelial cells. Pathway activity was quantified using AUCell scores based on curated KEGG/Reactome metabolism‐related gene sets. The x‐axis indicates the fold change in median AUCell activation score between CRC and healthy epithelial cells, and color represents −log10 nominal *p* value. Pathways were selected as exploratory top‐ranked pathways based on nominal significance and effect size. (G) Spatial Visium map highlighting tumor and epithelial cells. (H) Leiden clusters shown in UMAP space (left) and spatial coordinates (right). (I) Violin plot of malignant scores across Leiden clusters. (J) Violin plot of epithelial scores across Leiden clusters. (K) Spatial distribution of iron‐uptake activity scores. (L) Directionality of CCC. (M) Bar plot of metabolite‐mediated inter‐cluster communications, with iron‐mediated links highlighted. (N) Network visualization of iron‐mediated CCC (edges colored by MEBOCOST score). (O) Dot plot of communication strength between sender and receiver clusters. (P) UMAP of scATAC‐seq cell‐type annotations. (Q) Heatmap of differentially accessible chromatin regions in epithelial cells (CRC vs. healthy). (R) Heatmap of transcription‐factor enrichment in CRC versus healthy epithelial cells.

We next focused on metabolite‐mediated cell−cell communication (CCC). Myeloid and epithelial compartments were inferred to be among the most diverse senders and receivers (Supporting Information S1: Figure S1E,F). Across inferred metabolite interactions, iron and glutamine were predicted to be dominant mediators (Figure 2D), with iron signaling predicted to be associated with TFRC‐related communication from myeloid cells to epithelial cells. Consistently, *TFRC* and *SLC40A1* were upregulated in CRC myeloid and epithelial populations (Figure 2E). Within the Reactome “iron uptake and transport” gene set (R‐HSA‐917937; 56 genes), 32 genes (57.1%) were significantly increased in CRC (Supporting Information S1: Table S4), and pathway activity scores were elevated in CRC myeloid and epithelial cells (Supporting Information S1: Figure S1G). Notably, among all metabolite‐associated pathways, iron uptake/transport showed one of the largest CRC‐normal divergences (Figure 2F). Extending the analysis to microbiome−host interactions, we observed differential expression of *Enterococcus faecalis*‐interacting genes and enrichment for chemokine/cytokine signaling pathways (Supporting Information S1: Figure S1H), suggesting potential immune–microbiome coupling in CRC.

We then analyzed a published spatial transcriptomics dataset (3902 spots; 13,352 genes) using Ragomics‐Meta. Cell2location mapping of scRNA‐seq‐derived cell types onto the tissue suggested epithelial enrichment within tumor regions (Figure 2G). After standard preprocessing and Leiden clustering, we visualized cluster structure in both UMAP and spatial coordinates (Figure 2H–J), annotating clusters 1, 6, 7 as malignant and clusters 2, 4, 5 as epithelial. AUCell scoring of the Reactome “iron uptake and transport” pathway showed strong activation within tumor areas (Figure 2K,L). We further inferred spatial CCC with COMMOT and metabolite exchange with MEBOCOST, suggesting l‐glutamine and iron as potential key mediators of epithelial–tumor crosstalk, with malignant cluster 1 predicted to act as the primary signal receiver (Figure 2M,N). A global CCC‐strength dot plot was consistent with dense metabolic and ligand–receptor coupling between epithelial and tumor regions (Figure 2O).

Finally, we applied Ragomics‐Meta to scATAC‐seq data from CRC patients and healthy controls to investigate epigenetic and regulatory alterations associated with CRC metabolism. Cell‐type annotation identified major epithelial, fibroblast, and immune compartments (Figure 2P), and differential accessibility analysis revealed CRC‐associated chromatin accessibility changes in epithelial cells (Figure 2Q). TF motif enrichment further highlighted regulatory differences between CRC and healthy epithelial cells, including enrichment of TCF/LEF‐family motifs in CRC epithelial cells (Figure 2R). The overall scATAC‐seq workflow was summarized in Supporting Information S1: Figure S2A. After SnapATAC2‐based quality control, including assessment of unique fragment distributions, TSSe‐related quality metrics, and spectral embedding after filtering (Supporting Information S1: Figure S2B,C), we identified differentially accessible regions across Leiden clusters (Supporting Information S1: Figure S2D). An AI‐assisted interpretation module was then used to summarize the potential biological implications of motif‐enrichment results and their relevance to disease development (Supporting Information S1: Figure S2E and Supporting Information S1: Table S5). We further demonstrated LLM‐assisted pathway interpretation using a user‐provided gene list related to host–microbiome interactions and metabolic processes, illustrating the utility of Ragomics‐Meta for customized hypothesis generation (Supporting Information S1: Figure S2F). Integrating transcription factor motifs, regulatory regions, and putative target genes generated a TF‐centered regulatory network covering the top 50 features (Supporting Information S1: Figure S2G). The resulting TF‐regulated metabolism‐relevant gene list contained 196 genes with AI‐assisted functional annotations (Supporting Information S1: Table S6). KEGG pathway enrichment analysis of these metabolism‐relevant genes revealed multiple cancer‐ and metabolism‐associated pathways, including amino‐acid metabolism, glycolysis/gluconeogenesis‐related metabolism, and ion‐related pathways such as ferroptosis, supporting a potential role for metabolic and metal‐ion regulatory programs in CRC progression (Supporting Information S1: Figure S2H).

## 4 REPRODUCIBILITY AND FUNCTIONAL BENCHMARKING OF RAGOMICS‐META

To complement the visual UMAP comparison, we compared cell‐type annotations generated by marker scoring, CellTypist [10], SingleR [11] with HPCA reference, SingleR with Blueprint/ENCODE reference, and Azimuth/Seurat reference mapping. The major cell‐type compartments were largely consistent among marker scoring, CellTypist, and SingleR‐based annotations, whereas Azimuth/Seurat [12] showed relatively greater differences, possibly reflecting differences in reference composition and annotation granularity (Supporting Information S1: Figure S3).

We further quantified cross‐method concordance using pairwise Rand Index and Adjusted Rand Index. Marker scoring showed high agreement with CellTypist and SingleR‐HPCA, with Adjusted Rand Index values of 0.9216 and 0.8338, respectively. CellTypist and SingleR‐HPCA also showed strong concordance with each other, with an Adjusted Rand Index of 0.8587. In contrast, the agreement between marker scoring and Azimuth/Seurat was lower, suggesting that reference‐dependent mapping may introduce variation when the reference structure is not fully matched to the study dataset (Supporting Information S1: Figure S4).

To evaluate computational stability, we repeated the annotation procedures across four runs. CellTypist, SingleR‐HPCA, and SingleR‐Blueprint produced identical results across repeated runs, with a mean pairwise matching rate of 1.000 for each method. This supports the run‐to‐run reproducibility of the implemented annotation modules under identical input data and parameter settings (Supporting Information S1: Figure S5).

We also compared Ragomics‐Meta with representative single‐cell and omics analysis platforms, including Seurat [12], Scanpy [13], scTools, ezSingleCell [14], GranatumX [15], Partek Flow, Pluto, and Galaxy [16]. Ragomics‐Meta showed complementary advantages in multi‐format data input, standardized end‐to‐end workflow construction, cloud‐based analysis, AI‐assisted user interaction, reproducible workflow sharing, and metabolism‐oriented interpretation. This comparison highlights that Ragomics‐Meta is not simply a wrapper of existing algorithms, but a workflow‐level framework that integrates validated methods into a reproducible, extensible, and metabolism‐centered multi‐omics analysis ecosystem (Supporting Information S1: Figure S6).

Ragomics‐Meta represents a significant step forward in multi‐omics analysis, providing a unified, metabolism‐centered framework that facilitates the investigation of tumor biology across cellular and spatial contexts. By leveraging a modular design, it supports a more continuous analytical process, linking data processing, cross‐omics integration, interpretation, and reporting within a single workflow.

However, there are some important limitations to consider. First, while Ragomics‐Meta integrates multiple omics layers, the current integration is primarily conceptual and data‐driven, rather than a fully unified statistical model. Future versions of the tool may benefit from more advanced approaches that jointly model these modalities, providing deeper insights into complex biological questions. Additionally, Ragomics‐Meta's performance is dependent on the availability of high‐quality, well‐annotated datasets, and the tool's sensitivity to noisy data remains a challenge, particularly when working with newly generated or under‐annotated datasets.

The computational demands of Ragomics‐Meta, especially for large‐scale datasets like scRNA‐seq and spatial transcriptomics, require substantial computational resources. While divide‐and‐conquer strategies help mitigate runtime concerns, these requirements may limit accessibility for certain research labs. Furthermore, although Ragomics‐Meta has been successfully applied to ALL and CRC, its generalizability to other cancer types and diseases should be carefully validated.

Finally, the use of AI‐driven cell‐type annotation and result interpretation through large language models presents both opportunities and challenges. While these models enhance the tool's interpretability and functional annotation capabilities, their outputs should be validated against biological markers and known literature to ensure accuracy. Additionally, Ragomics‐Meta's fault‐tolerant design addresses most transient failures, but comprehensive failure‐case documentation is still in development.

## AUTHOR CONTRIBUTIONS

**Zeping Gui:** Conceptualization; supervision; writing—original draft; writing—review and editing. **Huidong Su:** Methodology; software, investigation; data curation; formal analysis; writing—original draft; writing—review and editing. **Yuhao Chen:** Methodology; software; writing—original draft; writing—review and editing. **Jixing Yuan:** Methodology; software; writing—review and editing. **Yexiu Gao:** Methodology; software; writing—review and editing. **Ze Yu:** Methodology; software; writing—review and editing. **Jing Zhang:** Investigation; data curation; formal analysis; writing—review and editing. **Qingyi Zhu:** Investigation; formal analysis; writing—review and editing. **Min Gu:** Supervision; writing—review and editing. **Mulong Du:** Conceptualization; supervision; writing—review and editing. **Zijie Wang:** Supervision; formal analysis; writing—review and editing. **Ming Chen:** Conceptualization; methodology; software; supervision; writing—original draft; writing—review and editing.

## CONFLICT OF INTEREST STATEMENT

Jing Zhang and Jixing Yuan are employees of RageneBio Inc., who developed the Ragomics platform utilized in this study. Yuhao Chen and Ming Chen contributed to the development of Ragomics platform during an internship, in a voluntary leadership role. Yexiu Gao contributed to the platform development in a software engineering internship. The remaining authors declare no competing interests.

## ETHICS STATEMENT

No animals or humans were involved in this study.

## Supporting information


**Figure S1:** Potential extensible explicability on gut metabolism activity and host‐gut microbiome interaction using scRNA‐seq data from CRC.
**Figure S2:** Analysis of CRC scATAC‐seq data offers extensive insights of tumour metabolism and regulatory mechanisms.
**Figure S3:** Multi‐method comparison of cell‐type annotation UMAPs and the ARI concordance matrix.
**Figure S4:** Quantitative concordance among cell‐type annotation methods.
**Figure S5:** Reproducibility assessment of automated annotation modules.
**Figure S6:** Functional comparison of Ragomics‐Meta with commonly used single‐cell pipelines and platforms.

**Table S1:** Data for human‐microbiome interaction genes.
**Table S2:** KEGG stored metabolism‐associated genes in 85 pathways.
**Table S3:** Reactome stored metabolism‐associated genes in 82 pathways.
**Table S4:** Differential expression analysis between CRC and healthy samples of the MSigDB human gene set enriched in “iron uptake and transport” pathway (Reactome: R‐HSA‐917937) using CRC scRNA‐seq data.
**Table S5:** Dialog between user and Ragomics Chatbot for transcription factor function and relation to disease development.
**Table S6:** CRC scATAC‐seq TF‐regulated metabolism‐relevant genes and brief function achieved by AI Chatbot.

## Data Availability

The website can be freely accessed at https://www.raganetwork.com/. For each case, data and analysis details can be accessed and downloaded at the following links: http://alpha.raganetwork.com/#/shared/982d5b3ccceaa62d6089e2ab7a0fe885f88c025a355cdb2fba212532d8334d90. The source code and relevant workflow scripts for Ragomics–Meta are available at GitHub: (https://github.com/Eggong/Ragomics-Metabolism). The datasets used in this study are publicly available from the following sources: ALL scRNA‐seq: (https://cellxgene.cziscience.com/collections/10ec9198-584e-4a7e-8a24-4a332915a4ef); CRC scRNA‐seq: (https://www.ncbi.nlm.nih.gov/geo/query/acc.cgi?acc=GSE132465, https://www.ebi.ac.uk/gxa/sc/experiments/E-MTAB-8410/results/cell-plots); CRC snRNA‐seq + scATAC‐seq: (https://www.ncbi.nlm.nih.gov/geo/query/acc.cgi?acc=GSE201349); CRC Spatial Transcriptomics: (https://www.ncbi.nlm.nih.gov/geo/query/acc.cgi?acc=GSE178318). The preprocessing steps for these datasets, including quality control, normalization, and dimensionality reduction, are detailed in the supplementary materials. The datasets are available for download and further analysis according to the respective data sharing policies. Supplementary materials (methods, figures, tables, graphical abstract, slides, videos, Chinese translated version and update materials) may be found in the online DOI or iMetaOmics http://www.imeta.science/imetaomics/.
