# Supplementary material for: Ragomics‐Meta: An open‐source, scalable, reproducible, and extensible multi‐omics analysis workflow
Source: IMetaOmics. 2026 Jul 17;3(3):e70121. doi: 10.1002/imo2.70121 (PMC13674391; doi:10.1002/imo2.70121)
Supplement: Supplementary file 1 [file IMO2-3-e70121-s001.docx]

**Supporting information to**

**Ragomics-Meta: An Open-Source, Scalable, Reproducible, and Extensible Multi-Omics Analysis Workflow**

**Running title：**

Ragomics-Meta for multiomics analysis

**Authors:**

Zeping Gui^1,2^*^#^*, Huidong Su^3^*^#^*, Yuhao Chen^2^*^#^*, Jixing Yuan^2^, Yexiu Gao^2^, Ze Yu^2^, Jing Zhang^2^, Qingyi Zhu^1^, Min Gu^1*^, Mulong Du^1,4*^, Zijie Wang^1*^, Ming Chen^2*^

^1^Department of Urology, Jiangsu Key Laboratory of Urological Disease Prevention and Treatment, The Second Affiliated Hospital of Nanjing Medical University, Nanjing Medical University, Nanjing, 210003, China.

^2^RageneBio Inc., Verona, New Jersey, 07044, USA.

^3^Department of Paediatrics and Adolescent Medicine, LKS Faculty of Medicine, The University of Hong Kong, Hong Kong SAR, 999077, China.

^4^Department of Biostatistics, Center for Global Health, School of Public Health, Nanjing Medical University, Nanjing, 211166, China.

*^#^*These authors contributed equally: Zeping Gui, Huidong Su, Yuhao Chen

*Corresponding: lancetgu@aliyun.com (Min Gu), [drdumulong@njmu.edu.cn](mailto:drdumulong@njmu.edu.cn) (Mulong Du), [njmuwzj1990@hotmail.com](mailto:njmuwzj1990@hotmail.com) (Zijie Wang), [chenming@raganetwork.com](mailto:chenming@raganetwork.com) (Ming Chen)

**Methods**

**Dataset-specific preprocessing and analysis parameters**

For all case studies in this manuscript, we applied dataset-specific quality-control and preprocessing steps appropriate for the corresponding modality.

**ALL scRNA-seq (PMID: 34864916; CELL×GENE collection ID: 10ec9198-584e-4a7e-8a24-4a332915a4ef):** Cells with fewer than 500 detected genes, fewer than 1,000 counts, or mitochondrial content greater than 15% were excluded. Doublets were identified and removed using Scrublet. Total counts were normalized to 10,000 per cell and log-transformed. Highly variable genes were selected using a variance-stabilizing method. Batch effects were corrected using Harmony, and principal component analysis was performed on the top 50 PCs. Graph-based clustering (Leiden, resolution = 0.5) and UMAP visualization were applied. Differential expression analyses used an adjusted *p*-value < 0.01 and |log_2_ fold change| > 1.5.

**CRC scRNA-seq (PMID:32451460; GSE132465; E-MTAB-8410):** Similar QC thresholds were applied, with cells filtered for fewer than 300 genes, fewer than 1,000 counts, or mitochondrial content > 12%. Doublets were removed using Scrublet. Normalization, HVG selection, PCA, and batch correction were performed as above. Clustering used Leiden resolution 0.5. Annotation combined manual review, CellTypist, and LLM-assisted predictions.

**CRC snRNA-seq + scATAC-seq (PMID：35726067; GSE201349):** Cells with TSSe < 7 or peak count < 1,000 were removed. Doublets were identified via Scrublet. Dimensionality reduction used spectral embedding and UMAP; clustering was performed using Leiden (resolution = 0.5). Differentially accessible regions (DARs) were called with adjusted *p*-value < 0.01. TF motif enrichment and peak-to-gene linkage were used to infer regulatory associations.

**CRC spatial transcriptomics (GSE178318; GSM5387661; SRX11048616):** Spot-level QC removed spots with fewer than 200 genes or > 20% mitochondrial content. Data were normalized and projected onto UMAP coordinates. Cell2location mapped scRNA-seq annotations to spatial spots. AUCell scored pathway activity for metabolism-related programs. COMMOT analyzed cell-cell communication, and spatial visualization was performed using Squidpy.

**Metabolic pathway and microbiome functional analyses**

Metabolism-related pathway activities were evaluated using curated KEGG- and Reactome-derived gene sets listed in **Tables S2 and S3**. Before pathway scoring, gene sets were intersected with the expressed gene universe of each dataset, and pathways with insufficient representation after intersection were excluded from statistical testing. Unless otherwise specified, pathways with fewer than 10 detected genes or more than 500 detected genes were removed to reduce instability from very small gene sets and nonspecific signals from overly broad gene sets.

Pathway activity scoring was performed using scMetabolism and AUCell, depending on the modality and analysis context. For AUCell-based analyses, gene rankings were generated for each cell or spatial spot, and pathway activity was quantified as the area under the recovery curve for each curated gene set. For scRNA-seq and snRNA-seq data, pathway scores were compared between CRC and healthy samples within each annotated cell type. For spatial transcriptomics data, pathway scores were compared between tumor-enriched and non-tumor regions or across spatial clusters, as appropriate.

For each pathway-level comparison, statistical significance was assessed using a two-sided Wilcoxon rank-sum test unless otherwise specified. P values were adjusted using the Benjamini–Hochberg procedure to control the false discovery rate across the tested pathways within each analysis context. For analyses involving multiple cell types, FDR correction was applied across the pathway-by-cell-type testing space. Differential pathway activity was summarized using the median difference or fold change of pathway activity scores, together with adjusted P values. Top-ranked pathway plots were generated from the predefined KEGG/Reactome metabolism-related pathway collections according to adjusted P value and effect size, and were interpreted as exploratory pathway prioritization.

Metabolite-mediated cell–cell communication was inferred using MEBOCOST. To facilitate customized pathway exploration, Ragomics-Meta includes an LLM-assisted chatbot for user-guided metabolic gene-list curation and biological interpretation. The LLM-assisted module was used only to assist gene-list organization and interpretation of pathway results, not to determine statistical significance. Potential host–microbe associations were explored by cross-referencing host genes of interest with the curated microbe–host interaction resource gutMGene (**Table S1**). These associations were interpreted as hypothesis-generating links rather than direct measurements of microbial abundance, strain identity, metabolite concentration, or spatial co-localization.

**Software environment and package versions**

Analyses were performed in Python 3.10 and R 4.2. Key packages included: Scanpy 1.9.1, Anndata 0.8.0, , BBKNN 1.4.1, Scanorama 1.7, SnapATAC2 1.0.0[1], Scrublet 0.2.3[2], HarmonyPy 0.0.7[3]，Squidpy 1.1.5, COMMOT 0.1.3, Cell2location 0.7.2, CellTypist 0.3.2, MEBOCOST 0.1.1, scMetabolism 0.2.4, Cardinal 2.2.1, AUCell 1.16.0, Seurat 4.3.0. LLM-assisted annotation used GPT-4 (API) and DeepSeek v2 (local deployment) with prompt temperature = 0.7; outputs were manually reviewed and cross-checked against canonical markers.

**scRNA-seq quality control, doublet removal, normalization, and highly variable gene selection**

Ragomics-Meta computes standard scRNA-seq quality control (QC) metrics directly on an input AnnData object (Scanpy v1.9+). QC metrics are calculated in a single step using the sc.pp.calculate_qc_metrics function block, optionally with user-defined control gene sets (e.g., mitochondrial genes and/or spike-ins). The per-cell QC summaries include total counts, the number of detected genes, and the fraction of counts assigned to control genes; these metrics are stored in the AnnData object and inspected using sc.pl.violin to guide filtering. Unless otherwise stated, we applied the following QC thresholds: cells were retained if the number of detected genes satisfied 200 < n_genes < 6,000 and mitochondrial gene fraction < 15%.

To mitigate multiplet artifacts, we predicted doublets using Scrublet through the sc.ext.pp.scrublet function block and removed predicted doublets prior to downstream analysis. In addition to doublet removal, we filtered out low-quality/outlier cells based on abnormal QC profiles (total counts, detected genes, and mitochondrial fraction), consistent with the above thresholds.

After QC and filtering, counts were normalized to reduce variation due to sequencing depth using sc.pp.normalize_total with target_sum = 1e4, followed by log-transformation (log1p). Highly variable genes (HVGs) were selected using sc.pp.highly_variable_genes, retaining the top nHVG = 2000 genes with flavor = "seurat_v3". The resulting normalized expression matrix and HVG set were used for subsequent dimensionality reduction, integration, clustering, and downstream analyses.

**scATAC-seq data analyses**

The CRC snRNA-seq and scATAC-seq dataset was obtained from GSE201349. The scATAC-seq component was analyzed using the Ragomics-Meta scATAC-seq workflow implemented with SnapATAC2 v2.9.0. Cells with TSSe < 7 or peak count < 1,000 were removed, and predicted doublets were identified using Scrublet and excluded before downstream analysis. A chromatin accessibility matrix was constructed using 500-bp genomic bins with add_tile_matrix. The top 50,000 features were selected for downstream analysis, followed by spectral dimensionality reduction using 30 components. A k-nearest-neighbor graph was constructed with n_neighbors = 100, UMAP embedding was performed with min_dist = 0.5, and Leiden clustering was performed at resolution 0.5.

Differential accessibility analysis was performed either across Leiden clusters or between CRC and healthy epithelial cells, depending on the analysis context. For cluster-level DAR identification, each cluster was compared against all remaining cells using a two-sided Wilcoxon rank-sum test implemented in the SnapATAC2 (v2.9.0) workflow. For CRC versus healthy epithelial-cell comparisons, the analysis was restricted to cells annotated as epithelial cells. P values were adjusted for multiple testing across all tested regions using the Benjamini–Hochberg procedure. DARs were defined as regions with FDR q < 0.05 and |log2FC| > 1. For visualization, the top-ranked DARs were selected according to adjusted P value and effect size. When sample-level metadata were available, we additionally summarized accessibility at the biological-sample level and used pseudobulk comparisons as a sensitivity analysis to evaluate whether the major CRC-associated accessibility patterns were consistent across samples.

TF motif enrichment analysis was conducted using DARs from the corresponding comparison. The background set consisted of accessible regions from the same cell-type-specific tested universe and was matched, where possible, by GC content, region length, and average accessibility. Motif enrichment was tested using an over-representation framework, and P values were adjusted using the Benjamini–Hochberg method. Motifs with FDR q < 0.05 were considered enriched. For the CRC versus healthy epithelial-cell comparison, TF enrichment results were visualized as a heatmap showing differential motif enrichment between groups.

Peak-to-gene linkage analysis was used to connect differentially accessible regions with putative target genes. Candidate links were defined between regulatory regions and genes located within a predefined genomic window around transcription start sites. When matched gene-expression information was available, accessibility–expression concordance was evaluated using correlation-based association analysis. Linkage statistics were corrected for multiple testing using the Benjamini–Hochberg method, and the resulting TF–region–gene associations were used to construct the regulatory network. The TF-regulated metabolism-relevant gene list was then subjected to downstream functional enrichment analysis. LLM-assisted modules were used to summarize motif functions, pathway implications, and disease relevance, while all statistical testing and enrichment calculations were performed using the corresponding computational modules rather than the LLM itself.

**Spatial genomics data analyses**

The spatial transcriptomics data were downloaded from Li et al. [4], and the spatial metabolomics data were downloaded from Hu et al. [5]. For 10x Genomics Visium spatial transcriptomics data, raw count matrices and spatial coordinate information were imported and processed following the standard scRNA-seq preprocessing workflow implemented in Ragomics-Meta. Briefly, low-quality spots were filtered according to detected gene numbers and mitochondrial gene fraction, followed by total-count normalization, log1p transformation, highly variable gene selection, PCA, neighborhood graph construction, UMAP visualization, and Leiden clustering [6]. Unless otherwise specified, spots with fewer than 200 detected genes or mitochondrial gene fraction > 20% were removed, and Leiden clustering was performed at resolution 0.5.

To map cell populations inferred from scRNA-seq data onto spatial transcriptomics profiles, we applied cell2location [7] to integrate scRNA-seq-derived cell-type annotations with 10x Genomics Visium mRNA count matrices. Metabolic pathway activity in spatial spots was calculated using AUCell based on curated metabolism-related gene sets, including REACTOME- and KEGG-derived pathways. Spatial distributions of cell-type abundance, cluster identity, and pathway activity scores were visualized using the <sq :: pl :: spatialscatter > function block with Squidpy [8] as the backend. Spatial metabolomics data were visualized and analyzed using the R package Cardinal [9]. These spatial genomics and metabolomics modules allow Ragomics-Meta to link tissue-level localization, inferred cell composition, and metabolic pathway activity within a unified workflow.

**AUCell statistical note**

Pathway activity scores between tumor-enriched and non-tumor spots were compared using a two-sided Wilcoxon rank-sum test. Tumor spots (n = 1,850) had a median AUCell score of 0.65; non-tumor spots (n = 2,052) median 0.42; *p* < 0.001. These results complement the visual observations and support enrichment of iron-pathway activity in tumor-associated regions.

**Microbiome**-**host resource**

In the present study, potential host–microbe interactions in the colorectal cancer datasets were explored using the gutMGene curated database, which links gut microbial species and genera to human host genes based on experimentally validated or literature-supported associations. Differentially expressed host genes or genes within pathways of interest were extracted from scRNA-seq, scATAC-seq, or spatial transcriptomics datasets and cross-referenced against gutMGene to identify associated microbial taxa, considering only associations above the default evidence threshold to reduce spurious links. The resulting host genes were further annotated with pathway information to interpret immune or metabolic interactions. These host–microbe associations were integrated with transcriptional and spatial data to generate hypothesis-driven insights regarding microbial influence on host pathways, including iron metabolism and immune programs, while explicitly noting that they do not constitute direct measurements of microbial abundance, strain identity, metagenomic content, or spatial co-localization. All outputs were interpreted as suggestive, and the workflow provides a framework to prioritize candidate microbial-host links for further experimental validation.

**Batch integration**

Batch effect is a common issue in scRNA-seq studies, particularly when cells are sampled from different donors or sequenced by different technologies. If scRNA-seq data are analyzed without proper batch correction, it may lead to misleading conclusions, as the data will likely contain non-biological biases that distort the true biological signals. Ragomics-Meta offers a variety of built-in function blocks to address the multi-sample integration problem, including <*sc* :: *ext* :: *pp* :: *harmony integrate>*, <*sc* :: *ext* :: *pp* :: *bbknn>*, and <*sc* :: *ext* :: *pp* :: *scanorama integrate>*, each employing different algorithms as the backend.

**Dimension reduction, clustering and annotation**

The high dimensionality and sparsity of scRNA-seq data, which involves thousands of genes and a large number of cells, present a big challenge to be visualized in 2 or 3 dimensions. Dimensionality reduction aims to preserve the biological variation of the data while projecting it into a low-dimensional space. Initially, Principal Component Analysis (PCA) is used to summarize the expression of genes across the top 50 principal components. Subsequently, nonlinear dimensionality reduction methods, such as Uniform Manifold Approximation and Projection (UMAP), are applied.

Cell clustering aims to group cells into diverse macro-states that share similar functional or molecular profiles. These clusters correspond to biologically meaningful cell types. This process is a critical step in scRNA-seq data analysis. In our workflow, a graph-based clustering approach is used, which consists of two main steps: (1) graph construction and (2) graph-based community detection. Graph construction typically involves k-nearest neighbor (KNN) algorithm. Ragomics-Meta provides a wide range of clustering algorithms such as Louvain, Leiden and partition-based graph abstraction (PAGA), which identifies groups of cells with high similarity within the graph and assigns them to subgroups based on graph theory.

For the datasets without prior knowledge of cell states, we apply the *celltypist* :: *annotate* function block to label the cell groups automatically. We also manually annotate the clusters based on differentially expressed markers or by utilizing LLMs-assisted annotation techniques in our ALL case.

**Differential expression genes analyses**

We use <*sc* :: *tl* :: *rank genes groups>* to find the differentially expressed genes in each cluster. This function block provides different statistical methods like logistic regression, t-test and Wilcoxon-test together with *p*-value correction methods such as Benjamini-hochberg and Bonferroni. Results can be visualized by volcano plot and heatmap. Genes with differential expression (DEGs) were identified by the threshold of adjusted *p*-value less than 0.01 and absolute value of log_2_ fold change (logFC) greater than 1.5.

**Pseudotime analyses**

Pseudotime is a temporal measurement of a cellular dynamic process such as cell differentiation and disease progression. The pseudotime inference function blocks such as <*sc* :: *tl* :: *dpt>* and <*pal* :: *core* :: *run palantir>* can be seamlessly integrated into the current pipeline and used for downstream Markovian analysis. We compute the pseudotime based transition matrix using CellRank pseudotime kernel and visualize the random walk as well as the identified terminal states in UMAP space.

**Metabolic functional analyses**

To deepen the mechanistic insights into tumor-microbiota-metabolism interdependencies, Ragomics-Meta offers a suite of specialized and advanced modules designed to facilitate this exploration. Firstly, users can delineate metabolic activity by leveraging metabolites from publicly available databases such as KEGG and Reactome, utilizing metabolism-related genes. The input gene pool can be derived from DEGs or a user-provided gene list. To streamline this process, our pipeline incorporates a pre-trained LLM-based AI chatbot that assists users in efficiently identifying relevant genes, which can then be seamlessly imported into pathway analysis. Secondly, we have integrated and enhanced several widely used R packages for metabolic analysis, including AUCell and scMetabolism [10]. Beyond their original functionalities, our pipeline provides a comprehensive analytical framework that allows users to compare results generated from diverse approaches. Thirdly, to enable users to efficiently pinpoint genes enriched in metabolic pathways of interest, we have incorporated two gene-pathway datasets. These datasets, derived from metabolism-associated genes and their corresponding pathway annotations, are sourced from two well-known repositories of KEGG and Reactome. Finally, Ragomics-Meta curates an extensive database linking gut microbiota species or genera to their target host genes in *Homo sapiens* [11]. One can swiftly identify genes highly associated with specific microbial species of interest and investigate their expression profiles across cancerous and healthy samples.

**LLM-assisted annotation and interpretation**

To support cell type annotation and functional interpretation, Ragomics-Meta incorporates a large language model (LLM)-assisted module together with reference-based annotation tools. In this study, GPT-4 was accessed through the GPTCelltype R package (v1.0.0) and the online GPT-4 API. Prompts were constructed from the top ten differentially expressed genes per cell cluster identified by two-sided Wilcoxon tests and were designed to request concise, biologically interpretable cell-type annotations. To improve reproducibility and reduce stochastic variation, LLM generation was performed using fixed prompt templates under controlled parameter settings. DeepSeek v2, deployed locally, was additionally used for domain-specific text guidance and functional interpretation.

CellTypist (v0.3.2) was used independently to provide reference-based automated annotations. LLM-generated annotations were not treated as stand-alone ground-truth labels; instead, they were cross-validated against CellTypist predictions, canonical marker gene expression, pathway context, and manual expert review. For clusters with ambiguous or mixed identities, the LLM outputs were used as assistive evidence to refine, rather than replace, expert annotation.

To evaluate concordance and stability, manual annotations, GPT-4-assisted outputs, and CellTypist predictions were compared across datasets. GPT-4 annotations fully or partially matched manual annotations in more than 75% of major cell types, with partial matches often reflecting differences in annotation granularity, such as fibroblast versus stromal-cell classifications. Repeated GPT-4 queries using identical marker gene sets showed reproducibility exceeding 85%, with Cohen’s κ = 0.67 across versions. For each dataset, the most frequent annotation across five repeated queries was selected as the final LLM-assisted assignment. These results indicate that the combined LLM-assisted and CellTypist-based strategy provides a robust, semi-automated annotation framework while maintaining expert oversight and biological interpretability.

**Hardware Configuration and Performance Benchmarking**

To assess the scalability and performance of Ragomics-Meta, we conducted benchmarking tests on both local servers and cloud instances. These tests aimed to evaluate the platform's efficiency when handling datasets of varying sizes and the effect of different computational resources on runtime. The hardware configurations used for the benchmarks included both a local server and a cloud instance, each chosen for their relevance to real-world usage scenarios.

The local server used for the initial benchmarking was equipped with an Intel Xeon Gold 6230 CPU (20 cores, 2.10 GHz), 128 GB of RAM, and 2 TB of SSD storage. This setup was primarily used for testing the ALL dataset to evaluate the platform’s performance in data preprocessing, analysis, and visualization. For cloud-based benchmarking, a cloud instance was deployed on [cloud provider name, e.g., AWS, Google Cloud, Azure], utilizing an Intel Xeon Platinum 8259CL CPU (36 cores, 2.5 GHz), 256 GB of RAM, and 3 TB of SSD storage. The cloud instance provided the flexibility to dynamically scale computational resources, simulating the handling of large-scale data.

Benchmarking on larger datasets, particularly those with over 500,000 cells, demonstrated the scalability of Ragomics-Meta. For instance, a dataset with 500,000 cells was processed on the local server in approximately 18 hours using 4 CPU cores, whereas the same dataset was processed on the cloud instance in about 10 hours with 36 CPU cores. The cloud instance showed a clear advantage in processing speed, particularly in tasks such as data preprocessing and dimensionality reduction, where the parallel processing capabilities of the cloud significantly accelerated the workflow.

The ability to scale resources on the cloud instance allowed for efficient handling of data-intensive tasks, reducing the time required for clustering, differential expression analysis, and spatial transcriptomics. The cloud setup also allowed for easier management of larger datasets, with cloud storage solutions facilitating seamless access to data and sharing across distributed teams. This cloud-based infrastructure removed the limitations of local storage and enabled smooth handling of large data volumes.

**References**

1. Zhang, Kai, Nathan R. Zemke, Ethan J. Armand, Bing Ren. 2024. “A fast, scalable and versatile tool for analysis of single-cell omics data.” *Nature Methods* 21: 217-227. <https://doi.org/10.1038/s41592-023-02139-9>

2. Wolock, Samuel L., Romain Lopez, Allon M. Klein. 2019. “Scrublet: Computational Identification of Cell Doublets in Single-Cell Transcriptomic Data.” *Cell Systems* 8: 281-291.e289. <https://doi.org/10.1016/j.cels.2018.11.005>

3. Korsunsky, Ilya, Nghia Millard, Jean Fan, Kamil Slowikowski, Fan Zhang, Kevin Wei, Yuriy Baglaenko, et al. 2019. “Fast, sensitive and accurate integration of single-cell data with Harmony.” *Nature Methods* 16: 1289-1296. <https://doi.org/10.1038/s41592-019-0619-0>

4. Li, Ruoyan, John R. Ferdinand, Kevin W. Loudon, Georgina S. Bowyer, Sean Laidlaw, Francesc Muyas, Lira Mamanova, et al. 2022. “Mapping single-cell transcriptomes in the intra-tumoral and associated territories of kidney cancer.” *Cancer Cell* 40: 1583-1599.e1510. <https://doi.org/10.1016/j.ccell.2022.11.001>

5. Hu, Junyi, Shao-Gang Wang, Yaxin Hou, Zhaohui Chen, Lilong Liu, Ruizhi Li, Nisha Li, et al. 2024. “Multi-omic profiling of clear cell renal cell carcinoma identifies metabolic reprogramming associated with disease progression.” *Nature Genetics* 56: 442-457. <https://doi.org/10.1038/s41588-024-01662-5>

6. Traag, V. A., L. Waltman, N. J. van Eck. 2019. “From Louvain to Leiden: guaranteeing well-connected communities.” *Scientific Reports* 9: 5233. <https://doi.org/10.1038/s41598-019-41695-z>

7. Du, Xinhao, Qing Li, Zhenzhen Tang, Li Yan, Ling Zhang, Qiao Zheng, Xianghao Zeng, et al. 2022. “Alterations of the Gut Microbiome and Fecal Metabolome in Colorectal Cancer: Implication of Intestinal Metabolism for Tumorigenesis.” *Frontiers in Physiology* Volume 13 - 2022, <https://www.frontiersin.org/journals/physiology/articles/10.3389/fphys.2022.854545>

8. Palla, Giovanni, Hannah Spitzer, Michal Klein, David Fischer, Anna Christina Schaar, Louis Benedikt Kuemmerle, Sergei Rybakov, et al. 2022. “Squidpy: a scalable framework for spatial omics analysis.” *Nature Methods* 19: 171-178. <https://doi.org/10.1038/s41592-021-01358-2>

9. Bemis, Kylie Ariel, Melanie Christine Föll, Dan Guo, Sai Srikanth Lakkimsetty, Olga Vitek. 2023. “Cardinal v.3: a versatile open-source software for mass spectrometry imaging analysis.” *Nature Methods* 20: 1883-1886. <https://doi.org/10.1038/s41592-023-02070-z>

10. Wu, Yingcheng, Shuaixi Yang, Jiaqiang Ma, Zechuan Chen, Guohe Song, Dongning Rao, Yifei Cheng, et al. 2022. “Spatiotemporal Immune Landscape of Colorectal Cancer Liver Metastasis at Single-Cell Level.” *Cancer Discovery* 12: 134-153. <https://doi.org/10.1158/2159-8290.CD-21-0316>

11. Cheng, Liang, Changlu Qi, Haixiu Yang, Minke Lu, Yiting Cai, Tongze Fu, Jialiang Ren, Qu Jin, Xue Zhang. 2022. “gutMGene: a comprehensive database for target genes of gut microbes and microbial metabolites.” *Nucleic Acids Research* 50: D795-D800. <https://doi.org/10.1093/nar/gkab786>

**Supplemental figures**

**Figure S1. Potential extensible explicability on gut metabolism activity and host-gut microbiome interaction using scRNA-seq data from CRC.** (A) Violin plots of total expression counts in each sample. (B) Violin plots of gene counts per sample. (C) Feature plots for expression of marker genes and Leiden clustering result on UMAP plots. (D) PAGA plot to show the connectivity among manually annotated cell types. A greater node indicates more cells in the closure. The darker the edge between two nodes, the greater the connectivity between the two clusters. For clarity, unspecified cell clusters were not present. (E) Barplot of the number of the metabolic-mediated CCC events across cell types. (F) The networking of the strength of metabolic-mediated signal transduction between any two cell types is evaluated by MEBOCOST. (G) Comparison of AUCell activation scores for the Reactome “iron uptake and transport” pathway between CRC and healthy samples across annotated cell types. For each cell type, pathway activity scores were compared between CRC and healthy cells using a two-sided Wilcoxon rank-sum test, with P values adjusted by the Benjamini-Hochberg method across tested cell types. Boxplots show the distribution of pathway activity scores, and significance levels indicate adjusted *p* values. (H) Stacked violin plots of host-microbiome interacting genes between tumor and healthy samples in four CRC-related gut microbiome species.

**Figure S2. Analysis of CRC scATAC-seq data offers extensive insights of tumour metabolism and regulatory mechanisms.** (A) Illustration of sample collection and analytical process using Ragomics scATAC-seq module. (B) The plot for scATAC-seq data quality assessment. The y-axis refers to the TSSe score. (C) Spectral embedding plot for CRC scATAC-seq data, coloured by doublet score. The darker the colour, the higher the probability of being doublets. (D) Heatmap of differentially accessible regions in each cluster. DARs: Differential accessibility regions. (E) A demonstration of AI-assistant analysis interpretation. We use TF enriched analysis as an example. (F) An illustration of TF-related pathway analysis accomplished by an LLM-based AI Chatbot implanted in the platform. (G) TF networking covering the top 200 features, where green, purple, and orange indicate motif, region, and gene, respectively. Orphan nodes (i.e., less connections with regions and/or motifs) are removed for clarity. (H) The pathway analysis of TF-regulated genes in relation to the metabolic process in the KEGG Human 2021 database. These metabolism-relevant genes were identified by an LLM-based AI Chatbot. Ion-related pathways are labelled with red stars.

**Figure S3. Multi-method comparison of cell-type annotation results.** UMAP visualizations showing cell-type annotations generated by marker scoring, CellTypist, SingleR with the HPCA reference, SingleR with the Blueprint/ENCODE reference, and Azimuth/Seurat reference mapping.

**Figure S4. Quantitative concordance among cell-type annotation methods.** Pairwise Rand Index and Adjusted Rand Index values were calculated to evaluate agreement among marker scoring, CellTypist, SingleR-HPCA, SingleR-Blueprint/ENCODE, and Azimuth/Seurat annotations. Higher values indicate stronger concordance between methods.

**Figure S5. Reproducibility assessment of automated annotation modules.** Annotation procedures were repeated across four independent runs to evaluate run-to-run stability. Within-method self-consistency was measured by pairwise matching rates across repeated runs.

**Figure S6.** **Functional comparison of Ragomics‑Meta with commonly used single-cell pipelines and platforms.** Ragomics-Meta was compared with representative tools and platforms, including Seurat, Scanpy, scTools, ezSingleCell, GranatumX, Partek Flow, Pluto, and Galaxy, across workflow construction, data input, cloud-based analysis, AI-assisted interpretation, reproducibility, and metabolism-oriented analysis features.
